# 

**Published:** 1840-09

**Authors:** 


### THE WESTERN JOURNAL OF MEDICINE AND SURGERY:

EDITED BY

DANIEL DRAKE, M. D.

AND

LUNSFORD P. YANDELL, M. D.

PROFESSORS IN THE LOUISVILLE MEDICAL INSTITUTE.

---

NO. IX.—SEPTEMBER, 1840.

---

LOUISVILLE, KY.

PUBLISHED MONTHLY, BY PRENTICE & WEISSINGER,

PROPRIETORS AND PRINTERS.

1840.

*This Number contains 4 sheets—Postage under* 100 *miles* 6 *cents, over* 100 *miles* 10 *cents.*

